# Physical Fitness Levels among Colon Cancer Survivors with a Stoma: A Preliminary Study

**DOI:** 10.3390/medicina56110601

**Published:** 2020-11-10

**Authors:** Hiromi Nakagawa, Hiroyuki Sasai, Kiyoji Tanaka

**Affiliations:** 1 School of Nursing, Takarazuka University, 1-13-16 Shibata, Kita-ku, Osaka City, Osaka 530-0012, Japan; 2 Research Team for Promoting Independence and Mental Health, Tokyo Metropolitan Institute of Gerontology, 35-2 Sakae-cho, Itabashi-ku, Tokyo 173-0015, Japan; sasai@tmig.or.jp; 3 Institute of Health and Sport Sciences, University of Tsukuba, 1-1-1 Tennodai, Tsukuba, Ibaraki 305-8577, Japan; tanaka.kiyoji.ft@u.tsukuba.ac.jp

**Keywords:** colorectal cancer, survivors, stoma, exercise, physical fitness age

## Abstract

*Background and Objectives:* Stoma surgery is linked to reduced physical activity in colon cancer survivors and leads to decreased physical fitness, activity of daily living dysfunction, and poorer quality of life. However, few studies have investigated the physical fitness levels of colon cancer survivors living with stomas. This study aimed to compare the physical fitness levels of colon cancer survivors with stomas and healthy adults, assessing them in a variety of dimensions (e.g., strength and flexibility) and in terms of physical fitness age (PFA), an integrated index of overall fitness. *Materials and Methods:* The study population consisted of 17 colon cancer survivors with (stoma group) and 20 healthy adults without (control group) a stoma. Physical fitness was assessed using a battery of five tests: repeated back-and-forth steps, 30-s chair stand, chair sit-and-reach, grip strength, and single-leg balance with eyes closed. Respective performance values were converted into PFA, which was compared between the stoma and control groups. Fitness indicators were compared between groups by analysis of covariance, and PFA and chronological age (CA) by paired *t*-tests. *Results:* The mean ages (±standard deviation) of the stoma and control groups were 74.1 ± 7.9 and 73.5 ± 7.1 years, respectively. Colon cancer survivors with stomas had poorer lower limb muscular strength, endurance, and flexibility than controls. In the stoma group, the marginal mean (±standard error) PFA was calculated to be 82.5 ± 3.7 years, significantly higher than the CA and PFA of the control group (69.6 ± 3.9 years). *Conclusions:* Colon cancer survivors with stomas have lower physical fitness levels than healthy adults, with apparent deficits in lower limb flexibility, muscular strength, and endurance. Our findings demonstrated the need for exercise interventions in this population, focusing on these dimensions of fitness. However, our results should be corroborated by means of a larger-scale comparison in future studies.

## 1. Introduction

A stoma is an artificial opening on the abdomen that allows the removal of feces or urine out of the body and is typically created in the surgical procedures for various gastrointestinal or urinary tract diseases. At present, nearly 700,000 people are living with a stoma in Europe, and over one million in the USA, in parallel with the increasing morbidity owing to colorectal cancer [1]. Similarly, over 13,500 members of the super-aging society in Japan live with a stoma [2]. One recent systematic review revealed that colon cancer survivors who underwent a stoma surgery (ostomy) were less physically active than survivors who did not (odds ratio = 1.51, 95% confidence interval (CI) = 1.12–2.04) [3]. Likewise, an online survey of 425 stoma patients in the UK found that respondents engaged in less physical activity after their ostomy procedure [4]. Such physical inactivity after stoma surgery and the fear of parastomal hernia development during the postoperative period were identified as major barriers to exercise in a questionnaire survey of 2631 stoma patients conducted by Russell [5] in the UK.

Recent studies have found that exercise interventions can help improve cancer-related fatigue and quality of life (QoL) in colon cancer survivors [6,7]. For these reasons, the American Cancer Society recommends that colon cancer survivors aim to engage in a minimum of 150 min of physical activity per week and include strength training at least 2 days per week [8]. These findings prompted us to start work on developing an exercise program for colon cancer survivors. However, as almost no study has sought to comprehensively evaluate the level and components of physical fitness in colon cancer survivors living with a stoma, we decided that a preliminary study aimed at characterizing colon cancer survivors’ physical fitness level would be a necessary precursor to the development of such an exercise program.

This study aimed to assess the physical fitness level of colon cancer survivors living with a stoma and determine in which respects it was remarkably different from that of healthy adults living without a stoma by comparing the performance of the two groups in a battery of fitness tests. We hypothesized that patients with a stoma would be less physically fit than those living without one.

## 2. Materials and Methods

### 2.1. Study Design, Setting, and Participants

This study has a cross-sectional design. Participants were recruited from meetings of a support group for stoma patients; attendees were notified of the study details in advance. Participants who consented to physical fitness testing were divided into two groups: the stoma group, consisting of colon cancer survivors living with a stoma, and the control group, healthy older adults without a stoma who were visiting or working at a local welfare facility for older adults. Stoma participants were recruited via the support group’s website and newsletter. Control participant were recruited via information posted on bulletin boards in the welfare facility.

Individuals who gave written informed consent to participate were enrolled if they fully met the eligibility criteria. For the stoma group, participants should not be regularly receiving cancer treatment (e.g., chemotherapy and radiation therapy) at the time of fitness testing. Patients diagnosed with inflammatory bowel disease or rheumatism were excluded. Additionally, patients receiving hemodialysis and palliative care or patients with neurological deficit disorders were also excluded from participation in the study.

For the control group, participants should either be regular attendees of the welfare facility, who were not regularly receiving medical care or engaging in noteworthy exercise or sports activities, or facility employees. Candidates were excluded if the researchers deemed them incapable of completing all the tests safely, e.g., due to poor general health or cognitive or walking impairments.

The study was conducted from August 2019 to May 2020 at the meeting place of the patient support group in Osaka city. All participants gave their informed consent for inclusion before they participated in the study. The study was conducted in accordance with the Declaration of Helsinki, and the protocol was approved by the Ethics Committee of Takarazuka University Institutional Review Board (No: 2019-6, approved date: 27 May 2019).

### 2.2. Procedure

Prior to physical fitness testing, consenting participants were requested to complete the self-administered anonymous questionnaire (see below). Then, the stoma group completed an additional questionnaire to assess fatigue. Finally, participants completed the fitness tests and an assistant recorded their performance on a measurement form. The participants sealed this form together with their questionnaire(s) inside a designated envelope and dropped it in a collection box at the test site for later collection by the research team, so as to ensure anonymity.

### 2.3. Measurement Parameters

#### 2.3.1. Demographic and Stoma-Related Characteristics

The following items were assessed by a self-administered questionnaire: age, sex, comorbidity, stoma type, years since ostomy, and history of stoma-related complications (namely, parastomal hernia or peristomal skin complications) and pain (with a free-response field for the affected area if affirmed). Researchers measured participants’ height and weight (increment: 0.1 cm, 0.1 kg) and used these values to calculate their body mass index (BMI).

#### 2.3.2. Cancer-Related Fatigue

Cancer-related fatigue was assessed exclusively in the stoma group by means of the Cancer Fatigue Scale (CFS), an instrument developed by the Psycho-Oncology Division of Japan’s National Cancer Center Research Institute East [9]. Fatigue is defined as a “subjective symptom characterized by generalized weakness including physical and affective exhaustion”; it is estimated to affect approximately 30% to 80% of cancer patients and can occur at any time in the disease’s progression, not only at end of life [10]. The CFS is a simple questionnaire designed to evaluate fatigue in cancer patients. The scale is composed of three subscales—physical, affective, and cognitive—having maximum scores of 28, 16, and 16 points, respectively. These subscale scores are summed to calculate the total score, which can range from 0 (representing a completely fatigue-free state) to 60 points (maximum score). Fatigue is considered severe when the CFS total score exceeds the cutoff of ≥19 points.

#### 2.3.3. Physical Fitness

The following five physical and motor performance measurements were collected: (1) repeated back-and-forth steps as an agility index, (2) 30-s chair stand as an index of leg muscular endurance, (3) chair sit-and-reach as an index of static flexibility, (4) hand-grip strength as an index of muscular strength, and (5) single-leg balance with eyes closed as an index of static balance [11,12]. The measurement protocols utilized in the study are summarized in Table 1. We selected these five tests because of the following reasons: (1) our participants were fit enough to engage in activities of daily living, as well as additional leisure activities typical of middle-aged and older adults; (2) natural aging-related changes in physical fitness would be reflected in participants’ performance on the tests; (3) the tests could be performed safely and simply without any need for special equipment. One assistant was stationed at each testing booth to ensure participants completed tasks safely without falling. In addition, two wound ostomy and continence nurses were present on standby in case of stoma-related issues during testing.

Physical fitness score (PFS) and physical fitness age (PFA) were computed from these 5 measurements using the following equations. PFS = 0.395X_1_ + 0.126X_2_ + 0.06X_3_ + 0.101X_4_ + 0.044X_5_ - 11.9 for men and PFS = 0.413X_1_ + 0.115X_2_ + 0.037X_3_ + 0.146X_4_ + 0.037X_5_ - 11.7 for women, where X_1_ = repeated back-and-forth steps (number), X_2_ = 30-s chair stand (repetitions), X_3_ = chair sit-and-reach (cm), X_4_ = hand-grip strength (kg), and X_5_ = single leg balance with eyes closed (sec). PFA = 0.341CA −8.703PFS + 91.98 for men and PFA = 0.041CA −9.868PFS + 65.94 for women, where CA means chronological age (years). The PFA was adopted as a unified indicator capable of integrating several dimensions of physical fitness, such as muscle strength and endurance, flexibility, agility, and balance. This metric was adopted for two reasons: (1) PFA can capture several aspects of a person’s physical fitness in a single index, which is calculated by means of a statistically valid method (principal component analysis, PCA); (2) PFA can be used to indirectly compare colon cancer survivors living with a stoma with healthy older adults, in whom it is largely equivalent to the chronological age (CA) [13].

In accordance with the 1990 protocol of Tanaka et al. [14], the above equations were constructed in advance based on 100 middle-aged and older Japanese adults (age: 40–80 years), recruited by neighboring residents, who had completed the five fitness tests. Their performance data were subjected to PCA, and participants’ PFA was estimated based on the resulting first principal component score. The equations were developed with the intention of broad applicability, from healthy people to frail individuals in need of long-term care—based on participants’ performance in the five fitness tests in Table 1.

### 2.4. Statistical Analysis

First, group attributes were summarized separately for the stoma and control groups. Next, fitness indicators were compared between the stoma and control groups. Finally, PFA and CA were compared within the stoma group. The specific statistical techniques used are described below. Data were processed using SPSS ver. 27 for Windows with a significance level of 5%.

#### 2.4.1. Descriptive Statistics

Group membership (stoma vs. control) was adopted as the categorical variable and all other items (except fitness indicators) selected as the outcome variables. Between-group differences were assessed by means of unpaired *t*-test for normally distributed continuous variables, Mann–Whitney U test for non-normally distributed continuous variables, and χ^2^ test for categorical variables.

#### 2.4.2. Physical Fitness in Stoma Group vs. Control Group

Group membership (stoma vs. control) was adopted as the categorical variable while PFA and fitness indicators were selected as the outcome variables. Between-group differences were assessed by means of analysis of covariance adjusted for sex, age, and BMI. As a non-normal variable, single-leg balance with eyes closed was log-transformed before model entry. Performance values are presented as means with standard error (±SE).

#### 2.4.3. PFA vs. CA within the Stoma Group

Differences between PFA and CA were assessed within the stoma group by means of paired *t*-test. Greater divergence was interpreted as higher PFA with respect to CA.

## 3. Results

### 3.1. Participant Attributes

Participant attributes of the two groups are summarized in Table 2. The stoma group consisted of 17 stoma patients who had undergone a colostomy. The control group consisted of 13 regular visitors to a local welfare facility for the elderly, who were not receiving medical care or regularly engaged in exercise or sports activities, and 7 adults working in the facility itself. Every participant successfully completed the full test battery. Most participants in both groups were male (stoma: 11 men (64.7%) vs. 6 women (35.3%); control: 12 men (60.0%) vs. 8 women (40.0%)). Likewise, the two groups were comparable in age (74.1 ± 7.9 vs. 73.5 ± 7.1 years, respectively) and BMI (22.2 ± 3.4 vs. 24.1 ± 3.0 kg/m^2^). All patients included in the stoma group were diagnosed with colon cancer without comorbidities and had permanent stomas created by open surgery.

One participant reported being affected by parastomal hernia, and nine by peristomal skin complications. In the stoma group, two participants reported joint and muscle pain in the wrist, shoulder, knee, hip, and thigh; one affirmed peristomal pain. In the control group, two participants reported pain in the knee and hip. The stoma group’s CFS total score (mean ± SD) was 24.1 ± 10.3 points; eight members (47.1%) exceeded the ≥19-point cutoff, indicating severe fatigue.

### 3.2. Physical Fitness Indicators

Table 3 shows the comparison of physical fitness indicators between the groups. Performance was statistically indistinguishable between the groups for the single-leg balance with eyes closed, repeated back-and-forth steps, and grip strength tests, indicating comparable balance, agility, and upper limb strength, respectively. However, the stoma group performed significantly worse on the 30-s chair stand, an indicator of lower limb strength and muscle endurance (21.5 ± 1.4 vs. 28.4 ± 1.3 reps: *p* < 0.05), as well as the chair sit-and-reach, an indicator of flexibility (3.8 ± 1.9 vs. 11.3 ± 1.8 cm: *p* < 0.05). In terms of PFA, the stoma group was significantly older than the control group (82.5 ± 3.7 vs. 69.6 ± 3.9 y: *p* < 0.05).

**Figure 1:**
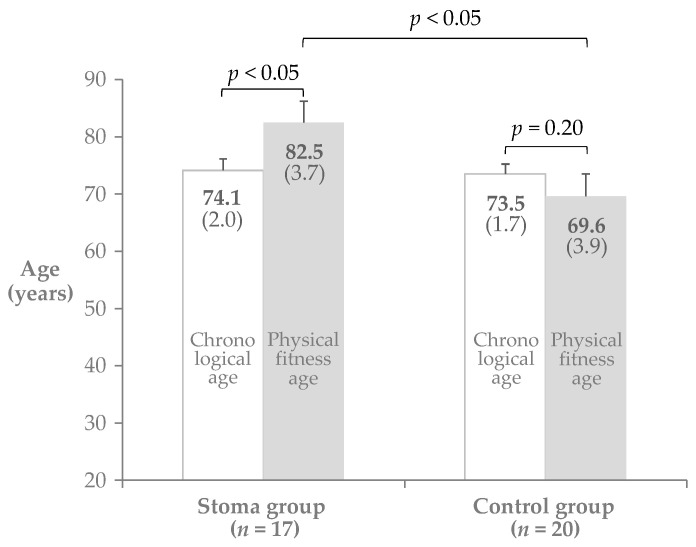
Physical fitness age (PFA) vs. chronological age (CA).

PFA was significantly higher than CA in the stoma group (82.5 ± 3.7 vs. 74.1±2.0, *p* < 0.05, 95% CI: −14.6, −2.2) but the two values were statistically comparable within the control group (69.6 ± 3.9 vs. 73.5 ± 1.7: *p* = 0.20, 95% CI: −2.3, 10.0) (Figure 1).

## 4. Discussion

The objective of this study was to assess the physical fitness level of colon cancer survivors living with a stoma and determine in which respects it was significantly different from that of healthy adults with no stoma by comparing their performance on a battery of fitness tests. We found that stoma recipients’ PFA was significantly greater than not only the PFA of healthy controls but also their own CA. In addition, stoma users’ fitness was noticeably poorer than that of healthy controls in terms of flexibility and muscular strength/endurance in the lower limbs, as evidenced by significantly lower scores on the corresponding fitness tests.

One potential explanation for the flexibility deficits measured in stoma patients during the chair sit-and-reach test is the presence of the stoma and appliance at the abdomen, restricting their ability to lean forward by contracting the abdominal wall. In addition, patients could have been fearful of parastomal hernia and intentionally avoided bending forward (or other movements to help them stay limber) to avoid increasing their intra-abdominal pressure (IAP) excessively. Parastomal hernia is a condition wherein a section of the small bowel or omentum starts to protrude through a stoma, causing the skin around it to “bulge” outward. They can be caused by surgical procedures or by patient factors, including increased IAP due to obesity or atrophy of the abdominal wall [15]. Previous research has noted that the fear of parastomal hernia after surgery was a major barrier to exercise engagement among stoma recipients [16]. These findings implicate parastomal hernia as a major contributing factor to poor physical fitness (hypodynamia) in this population. In addition, we discovered stoma patients to have poorer lower limb muscular strength and endurance than healthy controls, as measured by the 30-s chair stand test. We suspect that their physical activity levels were reduced by their need to continually wear an ostomy appliance—along with stoma-related complications, pain, and cancer-related fatigue—which impeded them from maintaining strength and endurance in their lower limbs.

Physical activity is a recommended therapeutic intervention for patients in chronic pain, which can help achieve functional improvements and alleviate pain and fatigue [17,18]. Exercise was found to reduce cancer-related fatigue and improve QoL in a systematic review of exercise interventions targeting colorectal cancer patients [3]; furthermore, it has been proven to effectively reduce recurrence risk and improve fatigue in patients being treated for colorectal cancer [19]. However, the key characteristics of such a program for this population—namely, the amounts, types, and intensities of component exercises—are still uncertain. This study was a preliminary study, the first step towards developing an exercise program optimized for colon cancer survivors living with stomas. Our results suggest that given their low fitness levels, stoma recipients should be assigned to exercise interventions aimed at improving muscular strength and endurance in their lower limbs after surgery, and that such exercise programs should be designed with parastomal hernia risk in mind.

Despite the high novelty of our work—the first reported comparison of physical fitness levels between colon cancer survivors living with a stoma and healthy individuals without one—there are a few limitations to this study. Firstly, we enrolled healthy individuals for our control (“non-stoma”) group; with the absence of serious disease, it is easy to imagine this population to have higher levels of physical fitness than colon cancer survivors. This decision also meant the groups could not be compared on the basis of cancer-related fatigue, as the CFS is not designed to be administered to healthy individuals. To address this disparity, further research must investigate levels of physical fitness and physical activity in colon cancer survivors who do not require stoma surgery. Causal explanations of the effects of having a stoma on physical fitness could be attempted based on a comparison of colon cancer survivors with and without stomas, allowing confounding interactions between stoma status and test performance to be isolated and eliminated. It was impossible to incorporate these into our statistical model because we were unable to properly measure them; even if it were possible, our sample size was restrictively small. Secondly, sampling bias was unavoidable as all colon cancer survivors living with a stoma were recruited via the same patient support group; caution should be exercised in generalizing our results. Given the high variability in physical fitness among stoma patients, future work must focus the investigation on a larger-scale study, applying the findings obtained in this study to develop suitable intervention programs.

## 5. Conclusions

This study aimed to determine the physical fitness levels of colon cancer survivors living with a stoma and compare them with those of healthy individuals living without a stoma, to determine in which respects they were remarkably different. Participants in the two groups did not perform noticeably differently on the single-leg balance with eyes closed, repeated back-and-forth steps, or grip strength tests, indicating comparable balance, agility, and upper limb strength. Stoma patients did exhibit worse muscular strength and endurance in the lower limbs, as well as flexibility, as evidenced by a significantly poorer performance on the 30-s chair stand and chair sit-and-reach, respectively. In addition, stoma users were found to have poorer fitness overall, with an estimated PFA higher than not only their own CA but also the PFA of healthy controls. Our findings suggest the need to develop exercise programs aimed at improving these dimensions of fitness in this population. To reinforce and validate the findings obtained in this study, researchers should conduct a large-scale survey—including colon cancer survivors who did not undergo an ostomy—with random sampling, to better ascertain the fitness levels of colon cancer survivors living with stomas and their contributing factors.

## Figures and Tables

**Table 1:** Fitness tests and measurement protocols.

Fitness Test	Measurement Protocol
Repeated back-and-forth steps	The participant stepped continuously in a constrained range—one step forward, one step backward—ensuring they placed their foot’s sole flat against the ground each time. The assistant counted the number of repetitions (1 forward + 1 backward) achieved in 10 s while monitoring the participant’s facial color and feet. The best of two attempts was selected for analysis.
30-s chair stand	The participant sat upright in a chair with a backrest with their arms folded across their chest. They were asked to stand up and sit down repeatedly, as quickly as possible, according to the assistant’s signals. The number of repetitions achieved in 30 s was recorded. The first (and only) attempt was selected for analysis.
Chair sit-and-reach	The participant sat upright in a chair with their shoes off, bent one leg at the knee (90°) with the foot flat on the floor, and extended the opposite leg with the heel on the floor (toes pointing towards the ceiling). They were instructed to reach forward, bending at the hip while slowly exhaling, and warned not to bend the extended knee (if so, the attempt was stopped). The assistant read the distance reached by the participant’s middle fingers as they paused for 2 s (increment: 0.5 cm). The best of two attempts was selected for analysis.
Hand-grip strength	The participant stood holding a grip strength meter (dynamometer) in one hand by their side and was instructed to grip the device as hard as they could while breathing normally. Grip strength was measured twice for each hand, alternating left and right (increment: 0.1 kg). The average of these four values was recorded for analysis.
Single-leg balance with eyes closed	The participant stood on one leg of their preference with their eyes closed, pressing their hands to their hips as they lifted the other foot above the floor, and was instructed to maintain this stance as long as possible (max time: 60 s). The assistant recorded the time elapsed from test start until the suspended leg touched the floor (or supporting leg) or one hand (or both) was removed from the hips. The best of two attempts was selected for analysis.

**Table 2:** Participant attributes by group: stoma vs. control.

	Stoma (*n* = 17)			Control (*n* = 20)			*p*
	Mean (SD) or *n* (%)		Min., Max.	Mean (SD) or *n* (%)		Min., Max.	
Sex (male)	11	(64.7)		12	(60.0)		0.44
Age (y)	74.1	(7.9)	57, 84	73.5	(7.1)	59, 85	0.79
Height (cm)	161.4	(7.7)	147.0, 173.0	161.3	(8.1)	147.0, 175.0	0.97
Weight (kg)	58.3	(12.3)	37, 80.6	62.8	(10.3)	39.2, 79.4	0.23
Body mass index (BMI) (kg/m^2^)	22.2	(3.4)	15.8, 28.3	24.1	(3.0)	18.1, 29.3	0.08
Years since ostomy	14.9	(14.3)	1.0, 40.0				
Postoperative complications							
Parastomal hernia	1	(5.9)					
Peristomal skin complications	9	(52.9)					
Pain							
Peristomal	1	(5.9)					
Systemic	2	(11.8)		2	(10.0)		
Cancer Fatigue Scale							
Physical subscale (pts)	8.7	(6.0)	0, 14				
Affective subscale (pts)	8.4	(2.9)	3, 19				
Cognitive subscale (pts)	7.0	(3.4)	1, 13				
Total score (pts)	24.1	(10.3)	9, 46				

Peristomal skin complications: abnormal changes to the skin around the stoma site—such as in coloration, shape, or structure—when compared to the abdominal wall skin. Standard deviation: SD; Min: Minimum; Max: Maximum.

**Table 3:** Fitness indicators by group.

	Stoma (*n* = 17)	Control (*n* = 20)	*p*
Repeated back-and-forth steps (reps/20 s)	16.1 (4.3)	16.4 (2.9)	0.94
30-s chair stand (reps)	21.5 (1.4)	28.4 (1.3)	< 0.05
Chair sit-and-reach (cm)	3.8 (1.9)	11.3 (1.8)	< 0.05
Grip strength (kg)	29.8 (2.6)	30.7 (2.2)	0.82
Single-leg balance with eyes closed (s)	27.1 (1.2)	40.4 (1.1)	0.09
Physical fitness age (years)	82.5 (3.7)	69.6 (3.9)	< 0.05

Estimated means are shown with standard error in parentheses. All models except for physical fitness age were adjusted for age, sex, and BMI.
